# A Framework for Biosensors Assisted by Multiphoton Effects and Machine Learning

**DOI:** 10.3390/bios12090710

**Published:** 2022-09-01

**Authors:** Jose Alberto Arano-Martinez, Claudia Lizbeth Martínez-González, Ma Isabel Salazar, Carlos Torres-Torres

**Affiliations:** 1 Sección de Estudios de Posgrado e Investigación, Escuela Superior de Ingeniería Mecánica y Eléctrica, Unidad Zacatenco, Instituto Politécnico Nacional, Mexico City 07738, Mexico; 2 Departamento de Microbiología, Escuela Nacional de Ciencias Biológicas, Instituto Politécnico Nacional, Mexico City 11340, Mexico

**Keywords:** optical biosensors, photonics, machine learning, nonlinear optics, SARS-CoV-2

## Abstract

The ability to interpret information through automatic sensors is one of the most important pillars of modern technology. In particular, the potential of biosensors has been used to evaluate biological information of living organisms, and to detect danger or predict urgent situations in a battlefield, as in the invasion of SARS-CoV-2 in this era. This work is devoted to describing a panoramic overview of optical biosensors that can be improved by the assistance of nonlinear optics and machine learning methods. Optical biosensors have demonstrated their effectiveness in detecting a diverse range of viruses. Specifically, the SARS-CoV-2 virus has generated disturbance all over the world, and biosensors have emerged as a key for providing an analysis based on physical and chemical phenomena. In this perspective, we highlight how multiphoton interactions can be responsible for an enhancement in sensibility exhibited by biosensors. The nonlinear optical effects open up a series of options to expand the applications of optical biosensors. Nonlinearities together with computer tools are suitable for the identification of complex low-dimensional agents. Machine learning methods can approximate functions to reveal patterns in the detection of dynamic objects in the human body and determine viruses, harmful entities, or strange kinetics in cells.

## 1. Introduction

The field of biosensors is highly dynamic, with scientific research advances that have mainly flourished in the last decades. Numerous biosensors have been developed for nanotechnology, engineering, molecular biology, computer, and optics [1]. In general, there are three types of biosensors: electrochemical, optical, and piezoelectric; each kind has its own method for transducing signals [2]. Nanoscale functions have been shown to be attractive for manufacturing biosensors [3].

A biosensor is a tool with the ability to detect and determine biological expressions in an environment [4]. This involves a biorecognition fragment for a detailed union and specifies the target molecules (enzymes, antibodies, proteins, cell receptors, toxins, DNA, pharmacists, etc.) [5]. Due to the powerful optical characteristics of semiconductors, they have provided great sensitivity and repeatability for integrated photonic biosensors based on silicon [6]. The performance of the optical sensors in semiconductor platforms may be impacted by two-photon absorption and free carrier dispersion, even if silicon offers optical advantages [7]. Therefore, different scientific groups have oriented their work to design other low-cost materials with advanced characteristics for developing optical biosensors [8].

Optical biosensors outperform standard analytical techniques by allowing real-time, label-free detection of biological and chemical compounds in a highly sensitive, selective, and cost-effective way [9]. Optical biosensors have been developed for detecting optical signals related to analytes via biocatalytic or bio-affinitive processes [10]. They are categorized according to the mechanism for biosensing, which can be refraction, reflection, Raman scattering, infrared emission, fluorescence, chemiluminescence, absorption, dispersion, or phosphorescence [11].

Optical biosensors can be assisted by plasmonic effects in order to easily identify a virus confirmed by molecules in exhaled air, or droplets such as those represented by nasopharyngeal swabs and saliva [12]. In essence, plasmonic detection techniques act as a viral pre-screening tool to enable the detection of infected individuals [13]. Biosensors have demonstrated their ability to detect viruses in human blood: an example is dengue [14] or chikungunya [15].

Surface plasmon resonance (SPR) has become the most sensitive label-free technique for the detection of various molecular species in solution, and it is of great significance in drug, food safety, and biological reaction studies [16]. SPR excitations are the result of free electron density oscillations and the interaction of electromagnetic waves between dielectric and metal film surfaces; the collective electronic excitations are the fundamental mechanism behind SPR experiments [1]. The reflected light in SPR systems is significantly reduced when the evanescent wave and the surface plasma wave produced by light resonate.

SPR technology has been utilized to produce biosensors for a variety of uses, including plasmonic detectors, optical polarization encoding, sensing technologies, and bio-photonic sensors [17]. SPR has been employed in several biosensor applications because it is highly sensitive to the refractive index of materials nearby [18]. The oscillation of free electrons in the conducting band of the metal is known as surface plasmons. They can only be excited by a polarized wave that is orthogonal to the plane of incidence and the direction of propagation of the surface plasmons [19]. Additionally, remarkable discoveries have been reported for biosensors based on the Raman effect, which is an inflexible shift in radiation frequency caused by optical light in vibrating molecules [20].

Plasmon-based technologies, such as SPR biosensors, have outstanding performance and versatility, and they are one kind of biosensor that is able to detect COVID-19 [21]. An SPR biosensor is capable of completing a reliable COVID-19 test in a matter of minutes compared to other long PCR or antigen tests that patients must perform in medical centers or hospitals [22]. Therefore, SPR-based techniques attract attention for developing biosensors. The detailed processing in SPR simply involves excitation of the coupled-resonator optical-waveguide at a fixed wavelength and imaging of coupled-resonator optical-out-of-plane waveguide’s elastic light-scattering huge factor [23]. The method can make use of a discontinuous transition of the coupled-resonator optical waveguide (CROW) eigenstate excited at a fixed laser wavelength in response to a slight change inside the coating refractive index [24].

Single protein detection has been achieved using several label-free optical techniques, including two with imaging capabilities. One involves heating a protein solution with a laser in an indirect manner while the change in the solvent’s refractive index is recorded. Interferometric scattering is the base of another technique [25]. The typical method for detecting the scattering light of plasmonic nanoparticles is based on scanning the spectra of nanoparticles using dark-field microscopy, which is time-consuming, laborious, and the small capacity of the sample regularly acts as a limitation [26]. On the other hand, surface-enhanced Raman scattering (SERS) methods are also assisted by SPR effects provided by specific metal nanoparticles such as their main component [20]. The double recognition biosensor SERS is an effective way to measure a variety of biological agents in the laboratory [27].

Biosensors based on bimetallic nanostructures have demonstrated high sensitivity in the detection of different substances, acting as an alternative for use [28]. In addition to their portability and high detection efficiency, some biosensors based on SERS can be reused more than three times when replacing the thread of the DNA substrate and washing the microfluidic device again [29]. Recently, several SERS substrates have been developed for biosensor applications with a high signal improvement for superimposed plasmonic fields. SERS is very attractive as an alternative method for detecting quantitative and co-multiplexed DNA because it can generate specific molecular oscillation spectra [30].

SERS -based methods have had a high impact on biomolecular analysis due to several factors, such as the fingerprint signal from the SERS nanotag and the stability [31]. As a rule, when a laser illuminates nanoparticles immobilized with the Raman reporter molecule, a local hotspot is initiated, and the Raman signal intensity of the reporter molecule is amplified by several orders of magnitude [32]. So far, research papers have been published demonstrating the potential of SERS-based methods for detecting sensitive and multiplexed biomarkers [33]. The dispersion of the cross-section spectrum shows a peak whose position also depends on the thickness of the biomolecular layer of the nanoparticles. The dependence of the cross-section spectra and the corresponding maximum changes in the thickness of the biomolecular layer are presented by a dispersing effect. Compared with the peaks of the absorption and dispersion spectra, the position of the peak of the dispersion spectrum is more sensitive to changes in the thickness of the biomolecule layer. The peak dispersion change can be about 8 nm, while the saturable absorption can be 2.5 nm [34]. In previous investigations, it has been pointed out that the optical characteristics of cadmium telluride nanorods have a better property under laser excitement with the absorption coefficient of two-photon absorption of 12.0 × 10^−10^ m/W at 100 μJ. Applications of cadmium telluride nanorods seems to be promising for the next-generation nonenzymatic biosensors and memory devices [35]. However, nonlinear optical (NLO) properties of semiconductors are limited by power level requirements. Nonlinear semiconductors are designed to exhibit high nonlinearity in refraction without effects associated with two-photon absorption; this method allows waveguides to operate at low power levels. For example, it has been indicated that silicon photon waveguide biosensors can detect variations in the transmission spectrum at 1550 nm of the urine glucose concentration with the evaluation of the refractive index [36].

It must be highlighted that NLO processes have opened up a variety of options for improving biosensors. There are many important factors to consider when designing nonlinear biosensors, including the refractive index of the optical media being used [37]. In particular, optical biosensors based on photonic crystals have been reported for detecting the concentration of the SARS-CoV-2 pathogen in water [38].

Moreover, in view of the need to overcome these issues, two branches of artificial intelligence (AI): machine learning (ML) and soft computing, have achieved a notable improvement in several research fields by providing agility and efficiency in different applications. Soft computing is an approach that incorporates the uncertainty and imprecision inherent to real world, inspired by systems in nature, mostly the human brain. Thus, a main process in these techniques is learning; machine learning, then, is related to the capability of a machine to infer an approximate solution from past data or to discover patterns and rules from unknown data.

In view of all these points, we analyzed different panoramic opportunities for optical biosensors based on NLOs for the detection of SARS-CoV-2. In this direction, we highlight how different NLO applications assisted by ML have increased their efficiency and speed to carry out tasks assigned to advanced algorithms with a potential for their use in sensing performance.

## 2. SARS-CoV-2 Biosensors

Compared to SARS-CoV and Middle East respiratory syndrome coronavirus, SARS-CoV-2 has been shown to be far more contagious [39]. The virus, also known as SARS-CoV-2, has had a significant negative impact on the environment and mankind, increasing mortality rates and causing significant economic losses around the globe [40]. In the years 2002 to 2003, the severe acute respiratory syndrome (SARS) was spread by SARS-CoV-2, a single-stranded RNA virus from the genus Beta coronavirus [41]. In 2021, RNA SARS-CoV-2 was frequently detected on surfaces in the medical environment, even in adaptive and unrelated sewage [42].

Coronavirus disease (COVID-19) outbreaks in several communities have compelled governments worldwide to enact stringent controls such as blockades, border closures, and widespread screening [43]. The SARS-CoV-2 virus is compatible with the coronavirus family with single-stranded gene RNA and surface proteins such as membranes, envelopes, nucleocapsids, and spikes [44]. Cryo-electron microscopy was utilized to establish the structure of the SARS-CoV-2 spike glycoprotein, which was then used for the creation of cell-specific vaccinations [45].

The symptoms of being infected by the SARS-CoV-2 virus can be varied; some symptoms are coughing, discomfort, and fever [46]. Several techniques are available for rapid measurement of antigen levels from both nasopharyngeal secretions and saliva, providing fairly satisfactory duplication of molecular assay results [47]. When performing the standard diagnosis, RNA extraction of the nasopharyngeal swab is required, followed by quantitative reverse transcription PCR (RT-QPCR) [48]. In recent years, some of the investigations have been focused on the design of optical biosensors for the efficient and rapid detection of the SARS-CoV-2 virus. The recognition elements of optical biosensors can be divided into aptamers, molecular imprint polymers (MIPs), and antibodies [49]. Wenjuan and his colleagues created the first unique microfluidic biosensor using Fresnel reflection for the detection of SARS-CoV-2 without a label that is quick, simple, and sensitive [50].

In order to identify the SARS-CoV-2 virus, optical biosensors can generate several wavelengths and collect data on heart rate, nitric oxide levels, pulse oximetry, and kidney function [51]. Courtney and colleagues created a successful biosensor with the ability to detect nucleic acids and with the option to improve with high convergence and mismatch [52]. Silicon nitride low-loss photonic wires have been used in the optical transmission waveguide devices to develop a complementary metal-oxide semiconductor compatible with the plasma-enhanced chemical vapor deposition process [53]. Ebola, HIV, and norovirus viruses have been detected by optical biosensors based on resonators, optical biosensors based on the waveguides, photonic biosensors based on crystals, and fiber-based optical biosensors [54]. In the latest investigations, the possibility of detecting the COVID-19 virus with a low 0.22 pm detection limit has been reported and the difference between SARS-CoV of the SARS-CoV-2 was distinguished by a plasmonic sensor [55].

One of the most intriguing and extensively researched devices is one made by utilizing surface nanopatterning technology. Nanopattern subwavelength characteristics promote actions such as guided mode resonance [56], SERS [56], or localized SPR [57]. Those structures make it possible to identify light interactions with certain biological analytes at the sensor surface effectively.

In order to increase the sensibility of sensing materials, photonic crystals have been proposed as periodic arrangements of dielectric materials built in an area of incoming radiation [58]. Similar to the bandgap in semiconductors, they have a photonic bandgap where it is forbidden for some wavelengths to pass through their structure [59].

In the past two decades, integrated photonic biosensors have become the focus of significant study because they can be miniaturized and can effectively detect relatively low concentrations of analytes in real time [60]. According to Srivastava and colleagues, the magnified changes caused by the conversion to photonics are sensitive to changes in the refraction index of the sensing medium; this makes the nanostructures an excellent choice for a biosensor [61]. Most of the photonic integrated sensors employ the concept of evanescent field detection, where the analyte adheres to a bioreaction layer on the surface of the wave guide and interacts with the evanescent field of the guided wave [62]. The initial displacement in particular biosensors may be increased by about four orders of magnitude by utilizing preselection to choose the polarization and postselection to create destructive interference [63]. This signal enhancement approach can simplify the optical components and lower the cost of the sensor device in addition to measuring the spin-dependent splitting in biosensors [64]. Furthermore, due to its distinct optical characteristics, the photonic spin Hall effect has generated a lot of study in recent years [62]. On-chip resonant or interferometric devices are used to translate changes in the optical phase, which cannot be detected directly, into changes in the optical power [63].

SPR biosensors are particularly effective in detecting bacterial viruses and pathogens among various biodetection methodologies [65]. By using this method, slow PCR and ELISA techniques are avoided. The first investigation by Wrapp and colleagues focused on the high affinity of the SARS-CoV-2 protein with ACE2 [66]. More recently, a unique localized SPR biosensor with the twin capabilities of plasmonic photothermal and sensing transduction was presented [67].

A very efficient technique for rapid detection is worth mentioning. It is without labels and is precise for a variety of pathogens and viruses that have been based on SPR [68]. In the past, it was asserted that an SPR-based biosensor could recognize the feline calicivirus in about 15 min [69]. In the same way, a very similar discovery was obtained for human enterovirus 71 (EV71) [70]. Research has found different forms of rapid and precise detection of COVID-19, and nanophotonic biosensors have been developed [67]. An SPR optical biosensor with a gold nanoparticle coating was successfully developed by researchers as a COVID-19 detection device [71]. For the potential detection of coronavirus illness, different optical biosensors with localized SPR have been presented [72].

It is possible to improve SPR platforms of localized SPR devices for the identification of COVID-19 [73]. Ren-min and Oulton’s study demonstrated the use of the nanolaser method as a biological optical detector [74]. For monitoring small chemical molecules, photonic glass fiber biosensors have been integrated by using porous silicon structures [75]. In order to find comparative chemical compounds, photonic crystal fiber biosensors based on a porous silicon have also been described [76]. Typically, the SPR biosensor is employed to identify biological or chemical materials [77]. Previous experiments demonstrated the potential of SPR biosensors for viral detection without real-time labels [78]. An overview of representative works in this area is shown in Table 1.

From Table 1, we can observe different optical and photonic biosensors that perform the function of detecting SARS-CoV-2. The advantage of using optical biosensors is the ease of use. The optics tools have demonstrated with some applications the ability to improve the resolution, speed, and efficiency of biosensors. Moreover, biosensors based on nonlinear absorption, Raman dispersion, or SPR can present advantages in biosensing regarding the potential for multiphoton effects. Table 2 presents these characteristics for detection of SARS-CoV-2. Table 1 describes biosensors assisted by optical effects, while Table 2 mentions biosensors that are related to multiphoton effects.

## 3. Biosensors Assisted by Machine Learning

As was mentioned before, ML is a subfield of artificial intelligence (AI) that provides another way to gain insight into complex data [137]. ML uses computational systems to simulate human learning and gives the algorithm the ability to recognize and acquire knowledge of the environment to improve performance [138]. Complex biological systems are naturally compatible with ML methods that can effectively detect hidden patterns [139]. Predictive information multiplexed can be obtained by increasing analysis of responses in a sequence [140].

**Figure 1:**
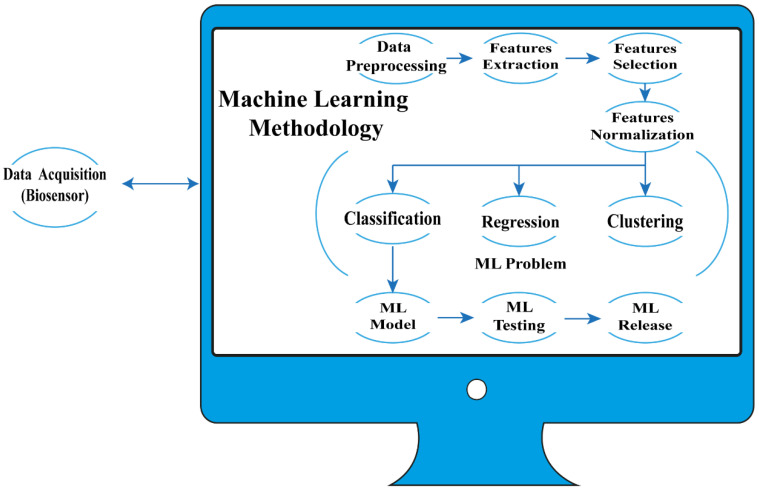
Biosensors assisted by ML.

ML-assisted biosensors can be used in complex environments and without having the characteristics of a laboratory study [141]. A typical process is shown in Figure 1. Raw data acquired by a biosensor are preprocessed (data filtering, missing values, segmentation; normalization is also carried out early in this step to homogenize scales or data types) according to the nature of the data. Features or characteristics are then extracted to represent the differences in the data and also to reduce the amount of data. This features set X is called features space. Dimensionality reduction of X is carried out to select the most significant variables and decrease complexity. It is worth mentioning that the quality of data is relevant. ML learns from the sample; if there is noise or the sample is not significant, overfitting will occur and the performance of the algorithm will be poor.

In general, three types of problems can be approximated with ML: classification, regression, and clustering problems. Dimensionality reduction by itself is also considered a type of problem solved by ML, and clustering is commonly a previous step in a classification problem.

**Figure 2:**
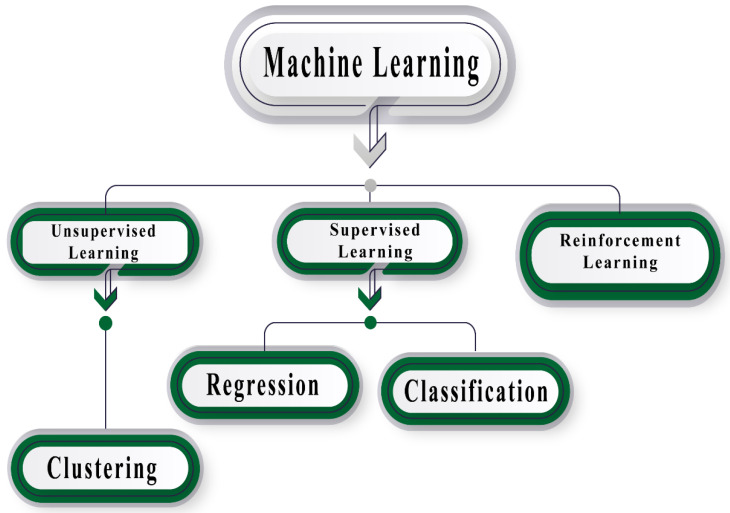
ML categories according to the nature of the features space.

According to the nature of X, the learning process in ML is divided into two main categories: supervised and unsupervised learning (Figure 2). When the inputs X are known or labeled, the learning process is called supervised. The objective in a problem of classification or prediction (regression) is to approximate a function f(X) = Y + ε, to approximate the output or labels Y with an error ε. In this learning process, ML methods use a subset of X to train a model. Once the model has been trained, it is tested with the rest of the available data. This step is repeated until the approximate function reaches an error goal; then, the model is released to classify or predict new unknown data. A balance among two types of error should be taken into account: bias, which is the result of the assumptions of data behavior in learning the objective function, and variance, which indicates how different the function approach will be according to the training dataset used. Different algorithms are used for these learning processes; some of them are usually applied to data analysis, such as linear regression. Other algorithms categorized in ML are logistic regression, support vector machines (SVM), naïve Bayes, decision trees, and k-nearest neighbors (KNN). On the other hand, the learning process is called unsupervised when X is not labeled; here, the objective is to discover the patterns in the data to generate clusters with similar features. The most popular algorithm for this learning process is k-means.

Soft computing algorithms are those strictly inspired by nature, for instance, artificial neural networks (ANNs), fuzzy systems, genetic algorithms, swarm algorithms, and ant colony optimization algorithms. Soft computing methods are especially useful for optimization problems; in this sense, ANNs and other ML algorithms optimize the error of the objective function.

The acquisition of information can be enhanced by automatic learning tools [142]; in this direction, optical biosensors have made a great contribution to medicine by being non-invasive and ultrafast. On the other hand, ML can improve these results, simplifying the analysis of the raw data from the biosensors output, to approximate a solution to different problems. For instance, (a) classification, for detection or diagnosis and treatment decisions support; (b) regression, to predict and prevent non-desirable events; and (c) clustering, to find groups of data that share features, such as symptoms, characteristics of a disease, or a strange behavior in different scales (e.g., enzymes, hormones, cells, organs, systems, and the whole body). The signals provided by the optical biosensor can be monitored in real time to outflow tract constructions that are useful in ML methods [143].

For instance, a supervised automatic learning method with optimized characteristics has been implemented to consider the effects of decreased enzymatic activity [144] or glucose in a sample [145]. ML regression statistical models have been applied to estimate the current response of a second-generation amperometry glucose oxidase biosensor [146].

### Neural Networks in Biosensors

**Figure 3:**
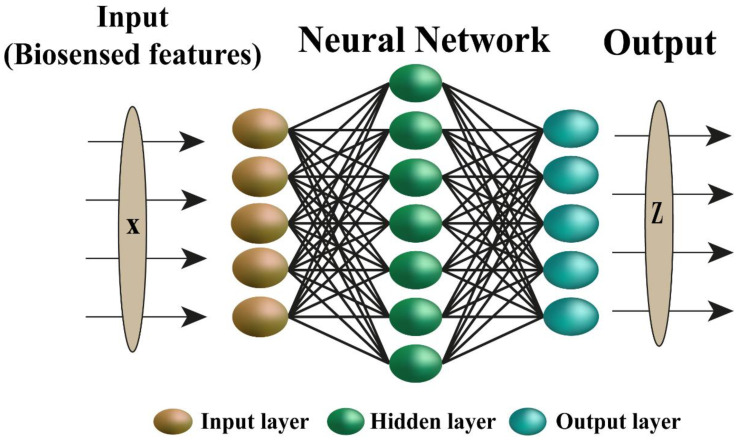
ANN common structure.

An artificial neural network (ANN) consists of a node layer that has an input layer, one or additional hidden layers, and an output layer [147]. Every node or artificial nerve cell connects to a different node and has acceptable weights and thresholds [148]. Once a private node output exceeds a threshold, that node is activated and sends data to a consequent layer within the network [149], as illustrated in Figure 3.

There is research demonstrating the improvement in the use of neural networks (NNs) in the enhancement of signal processing. In fact, it has been found that the combination of spectrum in spectrograms is an effective way to classify strong signs of biosensors [150]. In biosensors, pathogen agents and neurons associated with the disease have an important value. In recognition of the excellent classification capacity of the convolutional neuronal network model, it is also possible to perform the classification of a disease using biosensors [151]. An example of this is Mennel and colleagues, who conducted an image detection study applying an ANN [152].

In recent years, optical biosensors have received attention from the scientific community due to their advantages, such as detection with high sensitivity [153]. Different fluorescent materials such as quantum dots [154] and fluorescent microspheres have been used [155]. A technique to measure the fluorescent signal is excitation using a sensitive fluorometer; this determines the concentration of the bacteria. Instead of determining the target bacteria concentration, fluorescent bacteria can also be counted directly. NNs algorithms fulfill the function of processing the images obtained from fluorescent bacteria. NNs processing manages to calculate the amount of fluorescent points faster to determine the target bacteria [156].

## 4. NLO Processes Analyzed with ML

Prediction of nanoscale functions in multiphoton experiments is attractive for describing different NLO effects [157]. Analysis of third-order NLO techniques by ML has conjointly progressed throughout the last decade [158], considering all-optical functions for sensing and signal processing by ML [159]. There has been growing interest in generating pulses with repetitive frequencies on the order of gigacycle per second with the assistance of deep learning [160]. Measurements of ultrafast optical pulses for sensing represent challenges for scientific research in ML methods [161].

**Figure 4:**
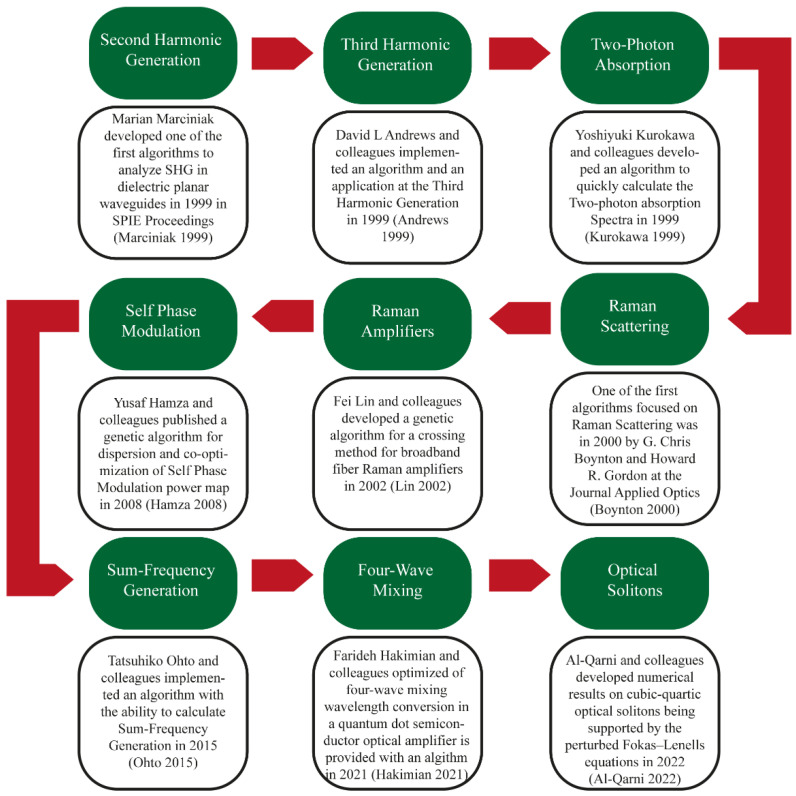
Roadmap of investigations based on NLO processes assisted by ML and soft computing [168,169,170,171,172,173,174,175,176].

A roadmap of representative research on NLO applying ML methods is shown in Figure 4. ML for studying chaotic nonlinear dynamics [162], self-tuning for mode-locked lasers [163], laser optimization [164], and the measurement of extremely short pulses; it should be noted that their duration is much shorter than the response times of most photodetectors [165]. Ultrashort pulses are widely used to monitor chemical reactions, control THz radiation, cipher pulses for communication, and form optical pulses [166]. ML has been used to measure time unit pulse duration using time unit detectors [167].

The most promising methodology to atone for nonlinearities in single channel systems is the digital backpropagation algorithm, which works by digitally modeling the fiber channel [177]. The disadvantages of this method are the high procedure complexity of the time period application and also the impossibility of accurately modeling the channel because of the looks of random parameters [178]. For these reasons, analysis on nonlinear compensation is currently centered on computing techniques [179]. Extraordinarily short pulses are troublesome to explain due to the massive variety of the parameters concerned [180]. With such systems, small changes in state variables will cause changes in momentum dynamics, which is particularly necessary with ML-based algorithms [168,169,170,171,172,173,174,175,176] (Roadmap).

### 4.1. Second-Harmonic Generation

The second-order NLO process in which photons that interact with a nonlinear material “combine” effectively is known as second-harmonic generation (SHG) [181]. SHG, which depends on a second-order NLO difference system, permits specialists to perform non-checking and non-horrendous imaging of tissue structures at the cell level [182]. Currently, when relevant areas in SHG images are detected, further medical actions can be proposed [183]. However, no simplifying assumptions or analytic solutions have been found to obtain SHG’s accurate spatial phase distribution [184]. The core measures employed in SHG simulation continue to be numerical techniques such as the split-step method and the Fourier-space algorithm [185].

The variation that uses SHG is simple for frequency-resolved optical gating (FROG). In fact, the pulse-shaping community frequently employs SHG FROG in nonlinear spectroscopy and coherent anti-Stokes Raman diffusing to discover potential extremely complex beats [186]. Furthermore, due to well-known trivial ambiguities, it has been mathematically demonstrated that all pulses may be uniquely predicted by SHG FROG [187]. More recently, a nonlinear time-domain finite difference method was developed by modifying Yee’s algorithm into a potent modeling technique that can take nonlinear phenomena such as second- or third-harmonic generation into consideration [188]. Intrinsic signals can be viewed as label-free using a nonlinear mode of multiphoton excitation called SHG [189]. Qun and colleagues have applied the SHG effect with the help of ML methods to develop images of the samples of thick heart tissue [190].

Since the discovery of quartz’s piezoelectricity more than a century ago, the need for effective materials for novel piezoelectric and NLO applications has steadily increased. Although piezoelectric materials are supposed to have the highest electromechanical coefficients, excellent SHG characteristics are crucial for NLO applications [191]. ANNs speed up optimization of genetic algorithms and store sample information that can be easily generalized to other samples with minimal additional training [192]. Hall and colleagues developed an impartial and efficient algorithm to quantify the images of SHG in tissues [193].

The continuous wave laser radiation in the UV range is often realized due to nonlinear effects such as four-wavelength mixing or SHG [194]. ANNs speed up optimization of genetic algorithms and store sample information that can be easily generalized to other samples with minimal additional training [195]. Deep-ultraviolet NLO crystals for current and upcoming basic research and technology requirements, a succinct SHG output wavelength, and a frequency conversion ratio are crucial [196]. SHG coefficients are shown to be inversely related to the band gap via the sum-over-states formula [197].

By using second-order NLO differential elements in SHG imaging, specialists can conduct label-free, non-destructive studies of tissue architecture [198]. Up to the current date, there is no published study that suggests using ML to instruct users about adjustable NLO vulnerability and exchanging behavior for sensing [199].

### 4.2. Nonlinear Optical Absorption

The optical absorption coefficient of a material that depends on irradiance is known as nonlinear optical absorption [200]. The absorption coefficient disappears at the dissipation intensity. In other cases, absorption is observed at low intensities, but the absorption coefficient increases or decreases at high intensities [201]. In order to address nonlinear tomographic absorption spectroscopy issues, Deng et al. looked deeper into how well other complicated deep ANNs, such as deep belief networks and recurrent ANNs, performed [202]. It is advantageous for photonic computing applications because of its straightforward design, very quick operation, and high NLO coefficient [203]. However, only basic investigations of direct absorption-spectroscopy-based deep learning algorithms for nonlinear tomography issues have been performed [204]. Only temperature or particle concentration may be reconstructed using the deep learning network provided [202].

### 4.3. Optical Kerr Effect

The Kerr nonlinearity has an NLO impact when light induces a change in the refractive index by different physical mechanisms such as electronic polarization or molecular orientation. It can be portrayed as an induced birefringence caused by optical irradiance and is dependent on the square of the electric field that can be supervised by ML [205].

The Kerr law of nonlinearity emerges when a light wave in an optical fiber meets nonlinear responses because of nonharmonic mobility of electrons trapped in molecules produced by an external electric field [206]. Solli and colleagues observed for the first time the rogue waves in one-dimensional settings in the field of optics [207]. Chalcogenide glass, which has a very strong Kerr effect and reacts right away to electrical stimulation, was employed by Gopalakrishnan and colleagues to obtain experimentally meaningful values for the above described [208]. Jhangeer and his colleagues developed an algorithm capable of obtaining wave solutions of exact paths of complex nonlinear partial differential equations [209]. This is achieved by improving the perturbative nonlinear Schrödinger equation with the nonlinear Kerr effect, which is an important equation for soliton testing in optical communication networks.

### 4.4. Sum Frequency Generation

A second-order NLO mechanism called sum frequency generation (SFG) works by annihilating two input photons with each frequency $\omega_{1}$ and $\omega_{2}$ while simultaneously generating one photon with frequency $\omega_{3}$ [210]. When imaging self-assembled thiol monolayers on gold using the SFG spectroscopic method, ANNs are utilized as a substitute for chemical identification [211]. ANNs are also particularly helpful for solving issues when it is difficult or impossible to provide realistic physical or mathematical models [212].

### 4.5. Self-Phase Modulation

An NLO result for the interaction between matter and the vectorial nature of light is self-phase modulation (SPM). Due to the optical Kerr effect, a medium’s refractive index changes when an ultrashort light pulse passes across it [213]. Since NN can adaptively correct for distortion, NN-based digital signal processing has been researched to account for nonlinear effects in wireless communication systems [214]. Only intensity-modulated direct detection transmission methods have been analyzed for nonlinear distortion correction in optical communication systems. In order to correct for the distorted multilevel optical signal caused by SPM, Shotaro and colleagues suggested a novel nonlinear equalization technique employing NN [215]. Caballero and colleagues developed a method with the ability to estimate signal-to-noise linear ratio and nonlinear ratio considering SPM assisted with an NN [216].

### 4.6. Raman Amplifiers

The reasonable choice of pump powers and wavelengths is a key element in accomplishing a wanted Raman pick-up profile. This is often a challenging assignment as the relationship between power profile versus pump powers and wavelengths is nonlinear and requires broad numerical reenactments to anticipate [217]. Raman amplifiers have lately attracted fresh interest as a result of their ability to amplify broadband signals by the assistance of ML when used in a multi-pump laser arrangement [218]. In addition, they have reduced noise when using distributed amplifiers and ML [219]. The Raman amplifiers’ capacity to arbitrarily set the gain by varying the pump power and wavelength is another distinctive quality improved by ML [220]. This gives optical amplifiers and optical communication systems unprecedented flexibility and capacity for dynamic adaptation by using deep learning techniques [221]. An example is distributed Raman amplifier (DRA), which is an important amplification method in optical communication systems due to its low noise figure and flexible wideband gain obtained by using ML [222]. Raman gain design and analysis have benefited greatly from the successful use of ML in other domains of optical communication in recent years [223].

Optimizing the pump design to obtain the appropriate gain spectrum at the amplifier output is the key research goal of the Raman amplifier [218]. This challenging optimization issue calls for the solution of a set of nonlinear differential equations. Many algorithms have been developed [224], as well as ANN [225] or ML [226] to find a solution to the conflict between the pump setting and the intended spectral gain setting. Currently, an ML strategy has been proposed for single-mode fibers [227] and few-mode fibers [225]. A dataset of hundreds of advantage bends made with erratic pump powers and wavelengths is used to train an NN to consider the relationship between the pump parameters [228].

### 4.7. Surface-Enhanced Raman Scattering

Molecular polarizability can be used to explain Raman scattering [229]. Electrons and nuclei are shifted when a molecule is put in an electric field [230]. An electric dipole moment is produced in the molecule because of the separation of charged species, and the molecule is said to be polarized [231]. A molecule scatters irradiant light from a source laser in the Raman method, which is a light scattering technique [232]. Most of the scattered light is of the same wavelength as the laser source and hence useless, but a tiny quantity of light is dispersed at various wavelengths and so is beneficial [233].

The molecules can be coherently driven to a state of breath and can then generate signals that are usually of a stronger magnitude than the spontaneous Raman dispersion [234]. This happens when there is a difference between the pump field and the Stokes field in the coincidence in active vibrations of the molecules in the sample [235]. By analyzing NLO effects, a quick and efficient response is required; an example is the impact of amplified spontaneous emission and nonlinear interference reported by Margareth and colleagues [236].

Raman microscopy is another option for label-free imaging; however, because of the poor effectiveness of Raman scattering, neuron imaging with ordinary spontaneous Raman scattering needs a considerable exposure period [237]. Plasmonic materials have been employed to boost the Raman technique’s sensitivity. Pengju and colleagues utilized a calculation based on ML to classify the ordinary and extracellular cancer vesicles and parties [238].

SERS, which has sensitivity down to the level of a single molecule, is perfect for multichannel detection [239]. Based on this idea, SERS physiology was very recently developed in order to offer speculative details about nearby cellular metabolites [240] by accumulating time-based SERS spectra constantly. The way the data were processed also had limitations in the original photophysiological trials for SERS. An ML method that is adaptable was proposed by Leong and colleagues [241].

### 4.8. Summary of Representative Nonlinear Optical Effects Assisted by ML Algorithms

The progress and development of new research in ML has opened up the opportunity to advance new techniques for the collection and interpretation of information in applications in different sciences. By joining the different optical processes to the interpretation of ML data, it opens up a variety of options and applications. The development of biosensors based on optical processes has provided the ability to detect biological agents in different organisms, facilitating their analysis. The study of the NLO processes assisted by ML involves the extraction of the properties that can represent fundamental information for sensitive classifying and segmentation.

NLO processes have been developed for the use of detection of different materials that can be used for improving biosensors. The most used multiphoton process for the detection of the SARS-CoV-2 virus has been Raman scattering; this is due to its advantages in the field of optics. Table 3 shows different NLO processes that are assisted by computer systems.

**Figure 5:**
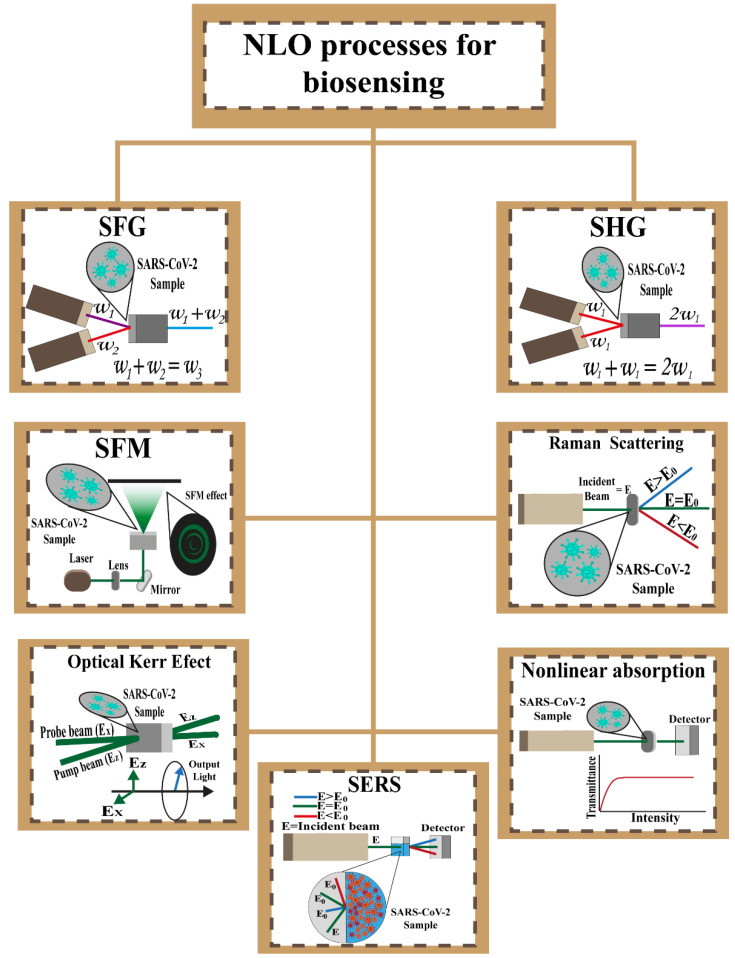
NLO effect schemes proposed for biosensing.

Table 4 shows different applications that improve the analysis of the processes of the NLOs assisted by computer systems. Figure 5 shows the different nonlinear optical effects mentioned in this work, with a sample of SARS-CoV-2 as an example.

## 5. Perspectives

Optical biosensors have evolved to visualize biological functions as a microscope has evolved to identify effects of energy. Some optical biosensors can focus the impact of optical and multiphoton nonlinearities to detect the SARS-CoV-2 virus. The optical effect of fluorescence has been used to identify the SARS-CoV-2 virus as well as bacteria, cancer cells, and other viruses. The signature of optics in biosensors has taken advantage of the collection of more information for the detection of biological agents as a non-invasive tool.

Due to the frequency and phase-changing capabilities of the laser light that interacts with NLO materials, they rank among the most intelligent materials of our time [264]. A cutting-edge topic of study for the theoretical and experimental community is the creation of NLO documents [279]. Organic materials must have relatively nonlinear properties due to electrons moving to orbits π−π [280]. This expectation explains the extensive research on NLO materials for developing biocrystals.

The use of organic crystals as NLO materials has been increasingly promoted by the easy manipulation of these crystals, allowing control of the NLO properties of the material. Compounds exhibiting strong nonlinearity are of great interest to the field of nonlinear optics, as they are used to fabricate devices operating at high speeds [281]. Researchers have been able to produce silicon-organic hybrid waveguides with bandwidths as high as 100 Gbit per second using organic NLO materials [282].

Optical biosensors can be applied to acquire information from remote sensing and one of the tools used for the interpretation of information can be based on ML. The function of the use of ML in biosensors allows automating the device to perform an action depending on the information collected. The diverse forms of emission and optical absorption in nonlinear biosensors are fascinating and are unexplored in several conditions that can be addressed by ML techniques for describing biological functions.

The disadvantage of AI derivatives is that there is a paucity of existing information on studies with NLO effects and nanomaterials, but this opens up an opportunity for new discoveries.

In the collection of information, different algorithms were found that analyze NLO effects. In Table 4, some algorithms are observed in different applications; NLO processes are unexplored for designing platforms related to biosensing performance, but they have promising potential.

## 6. Conclusions

ML has the potential to fundamentally change the practice of data analysis. Optical biosensors are well positioned to take advantage of ML, which leads to greater efficiency and precision. By combining the ML analysis tools and multiphotonic effects for the increase in applications in optical biosensors, it is clear that there is potential for a better interpretation of biological agents. In this work, a perspective for optical biosensors in virus detection is described.

The processing and classification of large amounts of data allowed by ML lead to extraordinary interpretations and unique predictions in the study with optical biosensors. In this work, optical biosensors assisted by ML for virus detection are proposed, specifically for SARS-CoV-2. By applying different NLO phenomena, the use of ML can optimize the biosensing performance due to its ability to handle large amounts of information. It was pointed out that there is still a vast field of research regarding the party effect of ML on nonlinear optical biosensors.

In this work, it is observed that ML can be useful for estimating different NLO interactions, although the current limited evidence does not support the superiority of ML and automation over study analysis in NLO processes. However, the handling and classification of large amounts of data allow envisioning that ML can play a crucial role in predictions of NLO-based biosensors. In this work, various studies that can be envisioned for the classification and organization of information in experiments with AI are proposed.

## Figures and Tables

**Table 1:** Representative optical biosensors papers for the detection of SARS-CoV-2.

Journal	Detection Limit	Analyte Types	Optical Effect	Year	Reference
*Biosensors and Bioelectronics*	2 μL	The genes of S, N, and Orf1ab	Evanescent wave fluorescence	2021	[79]
*Talanta*	1.0 mg/mL	Immunoglobulins (G, M, and A)	Colorimetric	2021	[80]
*Talanta*	12.5 ng/mL	IgG antibody	Evanescent wave fluorescence	2021	[81]
*Sensors and Actuators B: Chemical*	1 and 0.033 ng/mL	Spike 1 protein	Fluorescent bifunctional	2022	[82]
*Chemical Engineering Journal*	43.70 aM	RNA-dependent RNA polymerase gene	Electrochemiluminescence	2022	[83]
*Environmental Science: Nano*	32.80 aM	RNA-dependent RNA polymerase gene	Electrochemiluminescence	2022	[84]
*Biosensors and Bioelectronics*	2.75 fM	Spike protein, matrix protein, envelope protein, and nucleocapsid	Colorimetry G-quadruplex	2020	[85]
*Virology*	-	Nucleocapsid protein	Luminescence	2021	[86]
*Talanta*	59 aM	Nucleic acid	Electrochemiluminescence	2022	[87]
*Chemical Engineering Journal*	7.8 aM	RNA-dependent RNA polymerase gene	Electrochemiluminescence	2022	[88]
*Viruses*	50 μg/mL	Angiotensin-converting enzyme 2	Bioluminescent	2021	[89]
*Cold Spring Harbor Laboratory*	50 μg/mL	Angiotensin-converting enzyme 2	Bioluminescent	2020	[90]
*Physica Scripta*	1020 nm/refractive index unit (RIU)	Pathogens of SARS-CoV-2	Refractive index	2022	[38]
*Sensors and Actuators B: Chemical*	-	Spike protein	Optical interferometry	2021	[91]
*SSRN Electronic Journal*	833.33 nm/RIU	Spike glycoprotein	Refractive index	2022	[92]
*Talanta*	514 aM	spike protein, nucleocapsid protein, the RNA-dependent RNA polymerase gene	Electrochemiluminescence	2022	[93]
*Sensors*	0.1 fM	Open reading frames 1ab gene	Electrochemiluminescence	2022	[94]
*Talanta*	0.22 pM	Spike protein	Refractive index	2021	[55]
*Analytica Chimica Acta*	48 ng/mL	SARS-CoV-2 spike antigen	Colorimetric	2021	[95]
*Analytica Chimica Acta*	1.0 × 10^−6^ RIU	Spike protein receptor-binding domain	Fresnel reflection	2021	[96]
*2021 IEEE 15th International Conference on Nano/Molecular Medicine & Engineering (NANOMED)*	$114.07\;\mathrm{nm}\;{\mathrm{RIU}}^{-1}$	COVID-19 virus detection by delivering quick, dependable results	Refractive index	2021	[22]
*Scilight*	∼106 virions/mL	SARS-CoV-2 proteins (membrane, envelope, and spike)	Colorimetric	2021	[97]
*Biosensors and Bioelectronics*	17 aM	SARS-CoV-2 RNAs with single molecule sensitivity	Electro-optofluidic	2021	[98]
*Biosensors and Bioelectronics*	-	Nucleic-acid-based testing	Colorimetric	2021	[99]
*Journal of the American Chemical Society*	-	Spike antigen and cultured virus	Luminescent	2022	[100]
*Biosensors and Bioelectronics*	370 vp/mL	SARS-CoV-2 virus particles in one step	Nanoplasmonic resonance	2021	[101]
*ACS Applied Materials & Interfaces*	0.21 fM	RNA-dependent RNA polymerase gene	Electrochemiluminescence	2021	[102]
*In vitro models*	1 μg/mL	S protein of SARS-CoV-2	Colorimetric	2022	[103]
*Biosensors and Bioelectronics*	3 copies/μL	Two regions in nucleocapsid gene (N1 and N2 genes)	Fluorescence polarization	2021	[104]
*Biosensors and Bioelectronics*	1 mg/mL	Immunoglobulins G and M	Optical/chemiluminescence	2021	[105]
*Viruses*	100 pM	Spike proteins, nucleocapsid proteins	Fluorescent	2022	[106]
*Microchimica Acta*	$4.98\;\mathrm{ng}/{\mathrm{mL}}^{-1}$	Angiotensin-converting enzyme 2	Colorimetric	2021	[107]

**Table 2:** Representative multiphoton biosensors papers for the detection of SARS-CoV-2.

Journal	Detection Limit	Analyte Types	Optical Effect	Year	Reference
*IEEE Sensors Journal*	2.5 ng/mL	Nucleocapsid protein	Plasmonic fiber optic absorbance	2021	[108]
*Biosensors*	0.047 μg/mL	SARS-CoV-2 pseudovirus	Surface plasmon resonance	2022	[109]
*Biosensors and Bioelectronics*	$0.77\;\mathrm{fg}/mL^{-1}$	Spike protein	Raman scattering	2021	[110]
*Sensors and Actuators B: Chemical*	50 and 10 pfu/mL	Angiotensin-converting enzyme 2	Raman scattering	2022	[111]
*ECS Meeting Abstracts*	-	Antibodies to SARS-CoV-2	Surface plasmon resonance	2021	[112]
*Analytical Chemistry*	$45.6\;\mathrm{to}\;86\;\mathrm{ng}\;mL^{-1}$	Nucleocapsid protein	Plasmonics	2022	[21]
*Biosensors and Bioelectronics*	2 ng/spot	spike S1, spike S1 S2, and the nucleocapsid protein	Fluorescent plasmonics	2021	[113]
*Sensors*	4.2 μg/mL	Spike protein	Photonics	2021	[114]
*Analyst*	$12\;\mathrm{fg}\;mL^{-1}$	Spike S1 protein	Surface plasmon resonance	2022	[115]
*Biomedical Vibrational Spectroscopy 2022: Advances in Research and Industry*	-	Spike protein	Raman spectroscopy	2022	[116]
*Plasmonics*	152°/RIU	Spike proteins, membrane proteins, envelop proteins, and nucleoprotein	Surface plasmon resonance	2022	[117]
*Sensors*	250 μg/mL	Spike (S1 and S2) proteins	Surface plasmon resonance	2021	[118]
*IEEE SENSORS 2021*	8.34 ng/mL	Spike protein	Surface plasmon resonance	2021	[119]
*Biosensors and Bioelectronics*	1 μg/mL	Nucleocapsid antibody	Surface plasmon resonance	2022	[120]
*AIP Advances*	$54.04\;{\mathrm{RIU}}^{-1}$	Spike glycoprotein	Surface plasmon resonance	2021	[67]
*Analytical Chemistry*	-	Spike surface glycoprotein	Surface-enhanced infrared absorption	2021	[13]
*Matter*	10 PFU/mL	Spike glycoprotein and membrane protein	Raman scattering	2022	[121]
*ACS Applied Nano Materials*	200 PFU/mL	Spike proteins	Raman scattering	2022	[122]
*ACS Nano*	0.22 pM	RNA-dependent RNA polymerase	Localized surface plasmon resonance	2020	[123]
*Analytical Chemistry*	-	Angiotensin-converting enzyme 2	Surface plasmon resonance	2020	[124]
*Biosensors and Bioelectronics*	150 ng/ml	Detect SARS-CoV-2 nucleocapsid proteins	Localized surface Plasmon resonance	2022	[125]
*Analytical Methods*	200 μL	Spike and nucleocapsid proteins	Surface plasmon resonance	2021	[126]
*Sensors & Diagnostics*	10 RU	Spike protein	Surface plasmon resonance	2022	[127]
*Biosensors and Bioelectronics*	$2\;\times \;10^{11}$ particles/mL	Nucleocapsid phosphoprotein gene	Raman scattering	2022	[128]
*BioChip Journal*	1.02 pM	Antibodies against nucleoprotein	Surface plasmon resonance	2020	[129]
*Nanoscale Advances*	$4.5\;\mathrm{fg}/mL^{-1}$	SARS-CoV-2 spike protein	Raman scattering	2022	[130]
*Biosensors and Bioelectronics*	0.08 ng/mL	SARS-CoV-2 spike protein	Localized surface plasmon resonance	2020	[131]
*Analytical Chemistry*	4 mg/mL	SARS-CoV-2 spike protein	Surface plasmon resonance	2021	[132]
*Plasmonics*	$1\;\times \;10^{13}$ ${\;\mathrm{per}\;m}^{2}$	Thiol-tethered DNA of SARS-CoV-2	Surface plasmon resonance	2021	[133]
*Talanta*	0.046 ng/mL	SARS-CoV-2 spike protein	Raman scattering	2022	[134]
*Talanta*	$100\;\mathrm{pg}/mL^{-1}$	Measurement of SARS-CoV-2 antibody	Photonic resonator absorption	2021	[135]
*Talanta*	37 nM	SARS-CoV-2 spike glycoprotein	Surface plasmon resonance	2021	[136]

**Table 3:** NLO processes assisted by computational methods.

Journal or Conference Event	Application	Optical Effect	Year	Reference
*Sensors*	Optical biosensors supported by algorithms for rigorous monitoring and control in the identification of bacteria	Light Diffraction	2020	[242]
*PLOS ONE*	Comparison between Marquardt Algorithm vs. Newton Iteration Algorithm for biomolecular interaction process between antigen and antibodies or receptors	Optical Surface Plasmon Resonance	2015	[243]
*Scientific Reports*	Improves the image difference between normal tissues and tumors	SHG	2021	[244]
*BMC Cancer*	An independent predictive measure of metastasis-free survival in patients with invasive ductal cancer	SHG	2020	[245]
*SPIE LASE*	Enhanced Pulse Extraction Algorithm FROG used for geometry	SHG	2019	[246]
*Atmospheric Measurement Techniques*	Measures the error between CO and CO_2_ by nonlinear absorption and fluctuations in interference coefficients	Nonlinear Absorption	2013	[240]
*Journal of Lightwave Technology*	A scheme allowing the soliton comb to be determined under a specific pump scan, with an error of <8%, verified by experimental measurements	Optical Kerr Effect	2020	[247]
*SSRN Electronic Journal*	Tackling the effects of the intra-polarization self-phase modulation and inter-polarization cross-phase modulation	SFM	2022	[248]
*Optics Express*	Optimizes the pump wavelength	Raman Amplifiers	2020	[249]
*Optical Fiber Communication Conference (OFC) 2020*	Gains improvements for a few mode fiber amplifiers	Raman Amplifiers	2020	[250]
*Spectrochimica Acta Part A: Molecular and Biomolecular Spectroscopy*	Detection of quantity of chlorpyrifos in rice.	Raman Scattering	2021	[251]
*Food Chemistry*	Quantifies the systemic fungicide residues of Benzimidazole (Thiabendazole) in apples	Raman Scattering	2021	[252]
*ACS Nano*	Performs the measurement simultaneously from gradients, at least eight in vitro metabolites along with different cell lines	Raman Scattering	2019	[253]
*2021 IEEE International Conference on Big Data (Big Data)*	Improves rapidity in the inspection of the techniques of images of cellular and tissue pathology	Raman Scattering	2021	[254]
*2018 Cross Strait Quad-Regional Radio Science and Wireless Technology Conference (CSQRWC)*	Sorts different varieties of honey	Raman Scattering	2018	[255]
*In Proceedings of the 2021 IEEE 21st International Conference on Nanotechnology*	Label-free method for detection of protective anthrax antigens based on SERS	Raman Scattering	2021	[256]

**Table 4:** Publications about NLO processes assisted by ML.

Journal	Application	Algorithm	Nonlinear Optical	Year	Reference
*Journal of Lightwave Technology*	A standard for optical quality	A new approach to direct-learning-based pre-distortion using ANN	High throughput coherent optical	2020	[257]
*2017 International Conference on Orange Technologies (ICOT)*	Using a Computer-Aided Diagnosis (CAD) system, stem cells in the stratum basale are studied	Convolutional ANN	Third-harmonic generation	2017	[258]
*Optical and Quantum Electronics*	Optimizing the wavelength conversion for four-wave mixing in a quantum dot semiconductor optical amplifier	A fresh method based on ANN and genetic algorithms	Four-wave mixing	2021	[176]
*IEEE Photonics Journal*	Extrapolates helpful characteristics and details from the SERS signal	A novel approach to enhance SERS signals using principal component analysis as an ML approach	Raman scattering	2020	[259]
*Applied Optics*	Encryption scheme	A fresh nonlinear picture encryption method using the Fresnel transform domain’s Gerchberg–Saxton phase retrieval algorithm	Fresnel transform domain	2014	[260]
*Micromachines*	In optical micro-resonators, achieves high-fidelity harmonic production	Algorithm Broyden Fletcher Goldfarb Shanno	High-fidelity harmonic	2020	[261]
*APL Photonics*	A unique method for eliminating Cross-Phase Modulation (XPM) coherent artifacts in ultrafast pumping	XPMnet algorithm	Cross-phase modulation	2021	[262]
*IEEE Photonics Journal*	Showcases an optical phase conjugation photoelectric nonlinear compensation method	Complex-valued deep NN	Optical phase conjugation	2021	[263]
*Optics Express*	The deep residual network is used to forecast the Raman spectra of ice and water to detect the ice-water contact as an identification challenge	Deep-learning-based component identification for mixed Raman spectra	Raman scattering	2019	[264]
*Environmental Science and Pollution Research*	Examines the impact of the fungicide difenoconazole on the quality of rat sperm	Compare the effectiveness of three categorization algorithms	Raman scattering	2019	[265]
*IEICE Communications Express*	Improved performance in terms of bit error rate and error vector magnitude by effectively compensating for the nonlinear distortion brought on by cross-phase modulation	A cutting-edge digital signal processing method based on ANN for cross-phase modulation correction	Cross-phase modulation	2018	[266]
*Optics Communications*	Creates empirical physical formulations based on experimental evidence for the light-scattering amplitude response functions of nematic liquid crystals, which are intrinsically nonlinear	Layered feedforward ANN	Light-scattering	2011	[267]
*IEICE Communications Express*	Compensates nonlinear distortion in optical communication systems	A three-layer ANN	Self-phase modulation	2017	[268]
*Scientific Reports*	SHG coefficients of NLO crystals with different diamond-like features are being studied	Random forests regression	Second-harmonic generation	2020	[269]
*IEEE Journal of Selected Topics in Quantum Electronics*	A nonlinear activation function in a feed forward optical NN	Optical ANN	Electro-optic	2019	[270]
*2019 9th International Conference on Cloud Computing*	Provides scenarios that demonstrate the relationship between quantum computers and a light of light in the NLO	Algorithm assisting photonic operations	Four-wave mixing and cross-phase	2019	[271]
*Chemical Communications*	Ratiometric analysis is used to provide a model for the prediction of the depth of two “flavors” of SERS active nanotags buried inside pig tissue	A proof-of-concept approach for the prediction	Raman scattering	2022	[272]
*International Journal of Optics*	Inference abilities for the task of classifying images	The deep NN with all-optical diffraction	Nonlinear diffraction	2021	[273]
*Advanced Photonics*	Enhancement of the third- harmonic generation in optimized metasurfaces and contributes to improving the amplitude of optomechanical vibrations	Deep learning techniques for the inverse design of nanophotonics	Third-harmonic generation	2020	[274]
*Conference on Lasers and Electro-Optics*	Performs image and audio classification	A universal algorithm for backpropagating	Second-harmonic generation	2021	[275]
*Optical Materials Express*	Activation functions for fully connected ANN, emulated in tensor flow	Photonic ANN	Induced transparency and reverse saturated absorption	2018	[276]
*Optik*	Encryption security has been improved to the greatest extent possible to fend off attempts	Modified Gerchberg Saxton Iterative Algorithm	Optical nonlinear cryptosystem	2021	[277]
*Optics and Lasers in Engineering*	Checks the security of a dual random-phase-coding-based nonlinear optical cryptosystem	chosen-plaintext attack algorithm and known-plaintext attack algorithm modifications	Based on double random phase encoding, the NLO cryptosystem	2021	[278]

## Data Availability

Data and materials are available upon reasonable request to C. Torres-Torres (ctorrest@ipn.mx).
